# Comparative Study of the Antibacterial Effects of S-Nitroso-N-acetylcysteine and Sodium Nitrite against *Escherichia coli* and Their Application in Beef Sausages

**DOI:** 10.3390/foods13152383

**Published:** 2024-07-28

**Authors:** Jingjing Guo, Zhiyi Li, Yujun Zhang, Xiaojing Tian, Lele Shao, Wenhang Wang

**Affiliations:** 1 Tianjin Key Laboratory of Food Quality and Health, Tianjin University of Science & Technology, Tianjin 300457, China; 2 College of Food Science and Engineering, Tianjin University of Science & Technology, Tianjin 300457, China; 3 College of Tea and Food Science and Technology, Anhui Agricultural University, Hefei 230036, China

**Keywords:** SNAC, NaNO_2_, pH responsive, *E. coli*, antibacterial activity, antioxidant

## Abstract

This study investigated the antibacterial effects of S-nitroso-N-acetylcysteine (SNAC) and sodium nitrite (NaNO_2_) against *Escherichia coli* and their application in beef sausages. Both SNAC and NaNO_2_ demonstrated pH-responsive antibacterial activity, with SNAC showing greater efficacy than NaNO_2_ (*p* < 0.05) at the same pH (3, 5, and 7). The reactive oxygen species (ROS) and reactive nitrogen species (RNS) induced in *E. coli* by SNAC were significantly higher than those induced by NaNO_2_ (*p* < 0.05), and both ROS and RNS values increased as the pH decreased. In addition, a lower pH led to more pores on the *E. coli* cell surface and increased membrane permeability, resulting in a more pronounced inhibitory effect. When applied to a beef sausage, SNAC-treated sausages had significantly lower total colony counts and carbonyl content compared to NaNO_2_-treated ones (*p* < 0.05). Consequently, SNAC shows great potential as a replacement for NaNO_2_ in meat products.

## 1. Introduction

According to World Health Organization (WHO) data, approximately 600 million people are infected by foodborne pathogens annually worldwide, resulting in up to 420,000 deaths [1]. Diseases caused by foodborne pathogens have become a global public health issue [2]. Meat and meat products are rich in proteins, essential amino acids, vitamins, minerals, and other nutrients [3], so they are highly susceptible to contamination by foodborne pathogens during processing, transportation, storage, and sale [4]. Preservatives, which play a crucial role in meat product preservation, are widely used in the meat industry due to their ease of use, low cost, and high efficiency [5] in effectively inhibiting the growth of microorganisms. Nitrite is a traditional preservative in the meat industry, serving multiple purposes [6]. Firstly, the nitroso produced by nitrite decomposition can react with myoglobin and hemoglobin to produce nitroso heme, which endows meat with a pink color and thus greatly contributes to the color of meat [7]. Secondly, as a metal ion-chelating agent, nitrite can bind the iron ions in hemoglobin to prevent iron ion degradation, thus exhibiting antioxidant properties [8]. Moreover, it can also improve the flavor and prevent rancidity of meat by inhibiting lipid peroxidation [9]. In addition, nitrite can inhibit the growth of microorganisms, such as *Bacillus cereus*, *Staphylococcus aureus*, *Listeria monocytogenes*, etc. [10,11,12], especially *Clostridium botulinum* [13]. As is well known, the botulinum toxin secreted by *C. botulinum* has a serious destructive effect on the human nervous system [10]. Nitrite can effectively inhibit the growth of *C. botulinum* spores [10,14]. However, the N-nitrosamine derived from nitrite has shown potential carcinogenic and teratogenic risks for the human body [15]. Thus, the safety issue of nitrite has always been controversial worldwide. In recent years, some studies have reported that the antibacterial effect of nitrite is closely related to the formation of nitric oxide (NO) from nitrite. Nitrite mainly produces NO through the enzymatic pathway (nitrite reductase, xanthine oxidoreductase, etc.) and the non-enzymatic pathway (gastrointestinal acid, ascorbic acid, myoglobin, etc.) to exert an antibacterial effect. Therefore, the conversion of nitrite into NO for bacteriostasis may reduce the production of nitrosamines to some extent.

The NO donor compound serves as a stable form for the storage and conveyance of NO, effectively addressing the challenges associated with NO, including its difficulty in transport and quantification, as well as its brief half-life duration [16]. NO donor compounds include N-Diazeniumdiolate (NONOate), inorganic metal nitrosyl complexes, S-nitrosothiols (RSNOs), organic nitrates and nitrites, etc. [17]. Among them, RSNOs show several advantages over other NO donors. Firstly, RSNOs have been demonstrated to exhibit low tissue and cell toxicity [18], as well as good bacteriostatic activity. For example, the skin scaffolds mentioned in one study were able to continuously release Cu^2+^ and catalyze the production of NO from RSNOs, which showed potent antimicrobial activity against the biofilms of *Escherichia coli* and *Staphylococcus aureus* by eliminating 79.8% and 79.3%, respectively [19]. Secondly, RSNOs have demonstrated considerable antioxidant efficacy in meat products. It has been demonstrated that S-nitroso-N-acetylcysteine (SNAC) exhibits superior antioxidant properties compared to nitrites under simulated industrial processing conditions without the adverse effects associated with nitrites, such as N-nitrosation, oxidation and nutrient loss [20]. Thirdly, RSNOs produce fewer carcinogens (N-nitrosamines) compared to nitrites. In a study by Adi Shpaizer et al., SNAC was found to produce significantly lower amounts of N-nitrosamines than other additives in both meat products and gastric juices [21]. Consequently, RSNOs are regarded as the most promising alternative to nitrites. In general, nitrite tends to decompose NO in acidic conditions, but RSNOs can decompose NO over a wider range of pH, thus showing a wider range of applications than nitrite.

The antibacterial mechanism of NO has been extensively investigated and is known to target multiple components within bacterial cells, including the cell membrane, DNA, and proteins. Likewise, the bactericidal effects of NO, attributed to oxidative and nitrosative stress, have garnered considerable interest among researchers. NO and the reactive oxygen species (ROS) and reactive nitrogen species (RNS) formed by it can cause significant cellular damage, including membrane disruption, protein oxidation, and lipid peroxidation in bacteria [22,23]. In addition, NO, as an extremely reactive molecule, can impede bacterial adhesion by interfering with adhesion-related proteins, which, in turn, suppress the activity of metalloproteinases that are crucial for bacterial respiratory metabolism [23]. Consequently, strategies that focus on converting nitrite into NO or on seeking alternative NO donors could potentially enhance bactericidal efficacy and minimize the formation of nitrosamines.

*E. coli*, as one of the most common foodborne pathogenic bacteria in meat and meat products, is prone to causing emesis, diarrhea, and other symptoms [24]. Likewise, *E. coli* has been used as a model bacterium for many bacteriostatic studies [25]. Sausages are among the oldest processed meat products known to humanity. To meet the diverse demands of consumers for meat products, a vast array of sausages is produced on a global scale. Nevertheless, sausages are susceptible to microbial contamination throughout the processing and storage phases [26]. In this research, the bactericidal effects and underlying mechanisms of SNAC and sodium nitrite (NaNO_2_) on *E. coli* under different pH (pH 3, 5, and 7) values were evaluated. Likewise, the potential application of SNAC as a bacteriostatic, antioxidant, and coloring agent in beef sausages was also investigated.

## 2. Materials and Methods

### 2.1. Material

N-acetyl-L-cysteine (at 98% purity) was purchased from McLean Biochemical Technology Co., Ltd., (Shanghai, China). The NaNO_2_ (purity ≥ 97%) was purchased from Tianjin Damao Chemical Reagent Factory (Tianjin, China). The sulfanilic acid (Griess I, 4 g/L) was purchased from Macklin Biochemical Co., Ltd., (Shanghai, China), and the N-(1(-Naphthyl) ethylenediamine dihydrochloride (Griess II, 2 g/L) was purchased from Jiangtian Chemical Technology Co., Ltd., (Tianjin, China). The other reagents were all of analytical grade.

### 2.2. SNAC Preparation

In brief, 1.63 g of N-acetyl-L-cysteine (0.01 mol) was taken into a beaker with 8 mL of ice water. Then, 2 mL of hydrochloric acid (1 mol/L) and 0.69 g of NaNO_2_ (0.01 mol) were added, and the reaction was stirred for 20 min in an ice-water bath to produce a red transparent solution, which was stored at −20 °C away from light.

### 2.3. Bacterial Strain and Bacterial Suspension Preparation

The *E. coli* (ACCC 11277) was purchased from the Agricultural Culture Collection of China (Beijing, China), and the strains were frozen in a −80 °C refrigerator. Following the procedures outlined by Tian et al. [27], the strains of the mid-exponential growth phase (6–8 h, OD_600_ = 0.4) were prepared. The obtained strains were rehydrated in a phosphate buffer solution (PBS, pH 7.4). The initial strain concentration was approximately 1 × 10^8^ CFU mL^−1^ for all treatments.

### 2.4. Sample Treatment

The pH of the *E. coli* suspension (1 × 10^8^ CFU mL^−1^) was adjusted to 3, 5, and 7, respectively. The SNAC and NaNO_2_ (1 mL) were dissolved in 20 mL of *E. coli* suspension and cultured at 37 °C for 12 h.

### 2.5. Determination of Inhibition Zone and Colony Counting of E. coli

#### 2.5.1. Inhibition Zone

In brief, 100 μL of *E. coli* suspension (1 × 10^8^ CFU mL^−1^) was plated on the surface of a Nutrient Agar medium. Aseptic Oxford cups were positioned on the Nutrient Agar medium’s surface. The antibacterial concentration of SNAC and NaNO_2_ was determined to be 600 mM by pre-experiment. The SNAC and NaNO_2_ (150 μL) dissolved in different PBS (pH 3, 5, and 7) were transferred into an Oxford cup, and the PBS without SNAC and NaNO_2_ was used as the control. Then, they were cultured at 37 °C for 24 h, and the diameter of the inhibition zone was measured.

#### 2.5.2. Colony Counting

The antibacterial activity of SNAC and NaNO_2_ was explored using the plate-counting method. The *E. coli* suspension was diluted 10 times by series, and 100 μL of the appropriate dilution was selected for plating on a Nutrient Agar medium and then cultured at 37 °C for 24 h for colony counting.

### 2.6. Determination of the NO Content in E. coli Suspension and E. coli Cells

Griess reagent was used to determine the cumulative release of the NO in an *E. coli* suspension and the content of NO inside *E. coli* cells. SNAC and NaNO_2_ (1 mL) were dissolved in 20 mL of *E. coli* suspension and cultured at 37 °C in a constant temperature incubator (DHP-9402, North and South Instrument Co., Ltd., Zhengzhou, China). At each time point (0.5, 1, 1.5, 2, 4, 8, 12, 24, 36, 48, and 60 h), 1 mL of bacterial solution was collected and added with 1 mL if Griess I and 1 mL of Griess II, followed by a 15 min incubation period in the dark. The absorbance was measured at 540 nm by a UV-visible spectrophotometer (UV 3600 Plus, Shimadsu Company, Kyoto, Japan).

The content of the NO inside *E. coli* cells was measured as follows: The *E. coli* cells were pulverized with an ultrasonic cell crusher (JY92IIDN, Ningbo Xinzhi Biotechnology Co., Ltd., Ningbo, China) and centrifuged at 4 °C, 8000× *g* for 10 min (TDZ5-WZ, Xiangyi Centrifuge Instrument Co., Ltd., Hunan, China). Then, 1 mL supernatant was collected and added with 1 mL of Griess I and 1 mL of Griess II, followed by a 15 min incubation period in the dark. The absorbance was measured at 540 nm by a UV-visible spectrophotometer.

The cumulative release of NO was calculated as Equation (1):

Cumulative release of NO (μM) = (A_540_ + 0.0015) × 156.25. (1)

A: absorbance.

### 2.7. Scanning Electron Microscopy (SEM) Analysis of E. coli Cells

The *E. coli* suspension was washed twice by centrifugation (8000× *g*, 15 min) with PBS (pH 7.4). The cell precipitate was fixed overnight with 5 mL of a glutaraldehyde solution (2.5%, *w*/*v*), and then washed twice by centrifugation (8000× *g*, 15 min) with PBS (pH 7.4). The cell precipitate was, respectively, dehydrated with different concentrations (10%, 30%, 50%, 70%, 90%, and 100%) of ethanol solution for 15 min. Subsequently, the dehydrated cell pellet was resuspended in anhydrous ethanol, and 10 μL of *E. coli* suspension was taken on the monocrystalline silicon sheet. The sample was then affixed to a specimen stub with conductive adhesive and observed by SEM (SU1510, Hitachi, Tokyo, Japan) at an acceleration voltage of 6.0 kV and a magnification of 10,000 times.

### 2.8. Determination of ROS and RNS in E. coli Cells

The *E. coli* suspension was washed twice by centrifuging at 8000× *g* and 4 °C for 10 min with PBS (pH 7.4). The cell precipitate was covered with a 500 μL of DCFH-DA probe (10 μM), incubated at 37 °C for 30 min in the dark, and then mixed every 10 min. The *E. coli* suspension labeled by the probe was washed twice by centrifuging at 8000× *g* and 4 °C for 10 min with PBS (pH 7.4), and then it was suspended in 1 mL of PBS (pH 7.4). The obtained *E. coli* suspension (5 μL) was observed by fluorescence microscope (BX53, Olympus Corporation, Tokyo, Japan) to measure the fluorescence distribution, and 200 μL of *E. coli* suspension was detected by a fluorescence enzyme spectrometer (Synergy HTX, BioTek Instruments, Inc., Winooski, VT, USA) to measure the fluorescence value. The excitation wavelength was 488 nm, and the emission wavelength was 525 nm.

The determination method of the RNS in *E. coli* was similar to ROS, where the probe used was an O_52_ probe (10 μM) with an excitation wavelength of 488 nm and an emission wavelength of 526 nm.

### 2.9. Fluorescence Staining of E. coli with Propidium Iodide (PI)

The *E. coli* suspension was centrifuged at 8000× *g* and 4 °C for 10 min with PBS (pH 7.4), and the cell precipitate was washed twice with PBS (pH 7.4). The obtained *E. coli* suspension was readjusted to OD_600_ = 0.1 with PBS. Then, 90 μL of *E. coli* suspension was added with 10 μL of PI (50 μM) and incubated at 37 °C for 15 min in dark. The stained *E. coli* suspension (5 μL) was observed by fluorescence microscope. The excitation and emission wavelengths were 535 nm and 615 nm, respectively.

### 2.10. Determination of Lipid Peroxidation (LPO) in E. coli Cells

The *E. coli* suspension was centrifuged (8000× *g* and 4 °C,10 min), and the supernatant was discarded. Then, 1 mL of the extract from the LPO detection kit was added to the bacterial precipitate, followed by the breakage of the *E. coli* cells using an ultrasonic cell crusher (JY92IIDN, Ningbo Xinzhi Biotechnology Co., Ltd., Ningbo, China). After centrifugation (8000× *g* and 4 °C for 10 min), the supernatant was collected and immediately chilled on ice for determination. Malondialdehyde (MDA) content detection kit (Beijing Solarbio Science & Technology Co., Ltd., Beijing, China) was used to detect the extent of lipid peroxidation of the *E. coli* cells. The unit of MDA content was mmol/10^4^ cell.

### 2.11. Application of SNAC and NaNO_2_ in Beef Sausages

#### 2.11.1. Beef Sausage Preparation

Fresh beef meat (striploin) was washed by sterile distilled water to remove the surface blood and impurities. Then, they were trimmed of fascia and cut into small cubes of an approximately 3 × 2 × 2 cm size. The cubes were then processed through a meat grinder at 18,000 rpm for 30 s, which was repeated three times to ensure uniform comminution. Subsequently, the comminuted meat was divided into three groups, with each group weighing 1 kg. The control was treated with 3% salt (3 g salt/100 g meat) and 50 mL of water for 30 min, and two experimental groups were cured for 30 min with the same amount of salt and either 50 mL of 0.146 mol/L SNAC or NaNO_2_ solution (equivalently 100 mg of NaNO_2_/kg meat) at 25 ± 1 °C. The collagen casings (approximately 16 mm in diameter) were pre-soaked in 60 °C water. Subsequently, the meat mixture was transferred into the casings using a manual rotary enema device (JCW-10, Hangzhou Bijie Technology Co., Hangzhou, China), and the ends of the sausages were sealed with a sausage fastener. Subsequently, the sausages were baked in an oven (90 °C, 30 min), after which they were steamed for 15 min to ensure that the internal temperature reached 72 °C. Subsequently, the sausages were cooled to 25 ± 1 °C. The sausages were placed in sterile disposable lunch boxes covered with cling film (Dizao Shunjie, Hebei Dizhao Plastic Products Co. Ltd., Hejian, China) and then stored at 4 °C. Samples were, respectively, taken at 0, 3, 6, 9, 12, 15, 18, and 21 days for subsequent analysis.

#### 2.11.2. Determination of Total Colony Count

Sausage samples (5.00 g) were weighed and mixed with 45 mL of 0.85% sterile NaCl solution in a sterile bag and homogenized by a homogenizer (FJ-200, Shanghai Taxidermy Model Factory, Shanghai, China) at 2500 rpm for 1 min. The initial 1/10 (*w*/*v*) dilution was followed by sequential dilutions. Appropriately, 100 μL of the sample dilution was plated on a plate count agar (PCA) medium and incubated at 37 ± 1 °C for 48 ± 2 h. Two parallel experiments were performed for each dilution. The storage was terminated when the total colony count exceeded 1 × 10^7^ colony-forming units/g (CFU/g).

#### 2.11.3. Determination of Carbonyl Content

In brief, 1.00 g of the sausage sample was accurately weighed and added with 10 mL of PBS (20 mmol/L, containing 0.6 mol/L NaCl, pH 7.4), followed by homogenizing with a homogenizer at 2500 rpm for 1 min. Two 0.5 mL portions of the homogenate were taken, and both were added to 2 mL of pre-cooled TCA solution (20%) to precipitate the proteins. The supernatant was discarded after centrifugation (10,000× *g*, 5 min). One portion was supplemented with 2 mL of 2 mol/L HCl that contained 0.2% 2,4-dinitrophenylhydrazine (DNPH) and another was supplemented with only 2 mL of 2 mol/L HCl as the control, and then both were left at 25 ± 1 °C for 1 h. Next, 2 mL of 40% TCA was added and the supernatant was discarded after centrifugation (10,000× *g*, 4 °C) for 5 min. The precipitate was washed 3 times with 2 mL of ethyl acetate-ethanol (v:v = 1:1), centrifuged again for 10 min (4 °C, 10,000× *g*), and then washed 3 times to remove excess DNPH. Next, 2 mL of guanidine hydrochloride containing 6 M guanidine hydrochloride (containing 20 mmol/L potassium phosphate, pH 6.5) was added and centrifuged for 2 min to remove insoluble substances (4 °C, 5000× *g*). The absorbance of the solution was determined at 370 nm using a microplate reader (Synergy HTX, BioTek Instruments, Inc., Winooski, VT, USA), and the carbonyl content was calculated with Equation (2):

(2) $$\text{Carbonyl content (nmol/mg protein)}=\frac{(A_{\mathrm{DNPH}}-A_{\mathrm{HC}1})\times 2}{0.5\times 22,000\times C\times D}\times 10^{6}\;\text{(C: mg/mL).}$$

A: absorbance, C: protein concentration, and D: cuvette diameter.

#### 2.11.4. Determination of TBARS

In brief, 3 g of sausage sample was placed into a 50 mL centrifuge tube. Next, 50 μL of 10% BHT and 20 mL of trichloroacetic acid was added accurately, and the obtained mixture was shaken at 60 rpm with a constant temperature of 50 °C for 15 min in a water-bath constant-temperature oscillator (WE-3, Tianjin Uno Instrumentation Co., Tianjin, China). Then, the mixture was filtered through a Whatman’s qualitative filter paper, and 5 mL of the filtrate was taken and added with 5 mL of 0.02 mol/L aqueous thiobarbituric acid, which was then heated at 95 °C in a water bath for 30 min. After cooling to room temperature, the absorbance value at 532 nm was measured by a microplate reader. TCA was used as a blank control, and 1,1,3,3-tetraethoxypropane was used as a standard. The TBARS values are expressed as milligrams of MDA per kg of sausage samples, as shown in Equation (3):

TBARS = (A_532_ + 0.004) × 2.476. (3)

A: absorbance.

#### 2.11.5. Determination of pH

A total of 3 g of the sausage sample was added to 30 mL of deionized water, which was then mixed thoroughly. The pH of the solution was determined using a pH meter (FE-28 Standard, Mettler Toledo Instruments Ltd., Shanghai, China), and the average value was taken from three replicates of each sample.

#### 2.11.6. Determination of Color

The redness value (a*) of the sausage was determined by a portable colorimeter (CM-700 d, Konica Minolta Holdings, Inc., Tokyo Japan) using a spherical geometry d/8°, a D_65_ light source, and a standard observer’s angle 10°, and the diameter of the opening was 8 mm. The instrument was calibrated with a white standard board before measurement, and the measurements were performed at any six locations inside the sausage.

### 2.12. Data Analysis

The results are reported as the average values and standard deviations (SD) of three replicates. The data of the inhibition zone and NO content at a pH of 7 were analyzed by T-test using SPSS 26.0 software (SPSS Inc., Chicago, IL, USA). The rest of the experimental data were analyzed by one-way analysis of variance (ANOVA) to analyze the impact of one factor when fixing other factors, where a total of three factors were present (control, SNAC, and NaNO_2_ treatment). A probability value of *p* < 0.05 was considered significant.

## 3. Results and Discussion

### 3.1. Inhibition Zone

**Figure 1:**
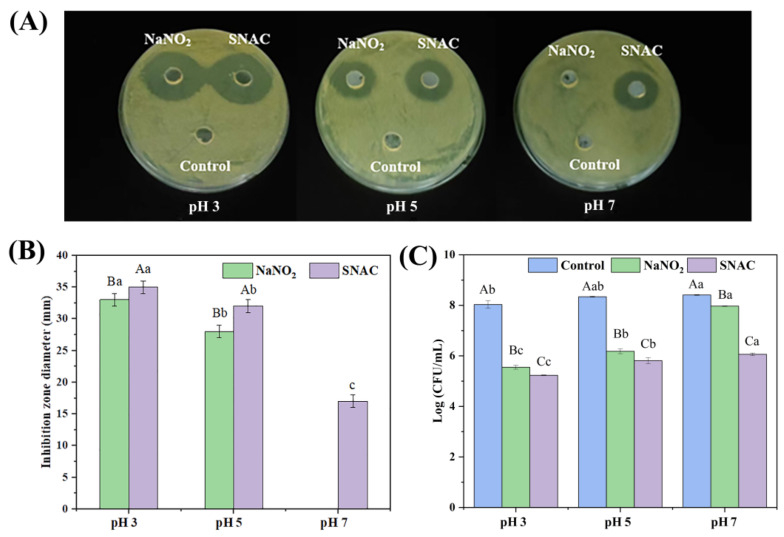
Visualization of the inhibition zone (**A**), diameter of the inhibition zone (**B**), and the colony counting (**C**) of *E. coli* by SNAC and NaNO_2_ at different pH values. The values are the means ± standard deviation of the triplicates. Uppercase letters indicate significant differences (*p* < 0.05) between different treatments at the same pH. Lowercase letters indicate significant differences among different pH values with the same treatment (*p* < 0.05).

The inhibition zone of SNAC and NaNO_2_ on *E. coli* is shown in Figure 1A,B. The diameter of the inhibition zone by both SNAC and NaNO_2_ was in the order of pH 3 > pH 5 > pH 7. Under pH 7, the antibacterial activity of NaNO_2_ was relatively low, which cannot be observed on the plate.

Compared with a pH of 7, SNAC and NaNO_2_ had stronger antibacterial activity under lower pH values, indicating that the antibacterial activity of SNAC and NaNO_2_ is pH-responsive. This might be attributed to the fact that more NO, as well as more reactive nitrogen and reactive oxygen intermediates, were produced under acidic conditions [28], contributing to its antibacterial activity. The inhibition zone of SNAC was greater than that of NaNO_2_ under the same pH, indicating that SNAC has greater antibacterial ability than NaNO_2_ [29].

### 3.2. Colony Counting Analysis of E. coli

The colony-counting results of the *E. coli* are shown in Figure 1C. Compared with the control groups, the colony counting of *E. coli* showed a significant decrease after SNAC and NaNO_2_ treatment (*p* < 0.05) under pH values of 3, 5, and 7, and the antibacterial capacity was in the order of pH 3 > pH 5 > pH 7 for both SNAC and NaNO_2_. The lower the pH, the better the antibacterial ability, which can be explained by the greater derivation of the active intermediate compounds from SNAC and NaNO_2_ in acidic conditions, thereby inhibiting the growth of *E. coli* [28]. Previous studies by Du et al. [30] gave similar results; they investigated NO production from nitrite in the pH range of 2.4–7.4, and the results showed that, at lower pH, nitrite produces NO faster. The antibacterial effect of SNAC was better than that of NaNO_2_ under a pH of 3, 5, and 7, which is consistent with the results of the inhibition zone.

### 3.3. Release Behavior of NO

**Figure 2:**
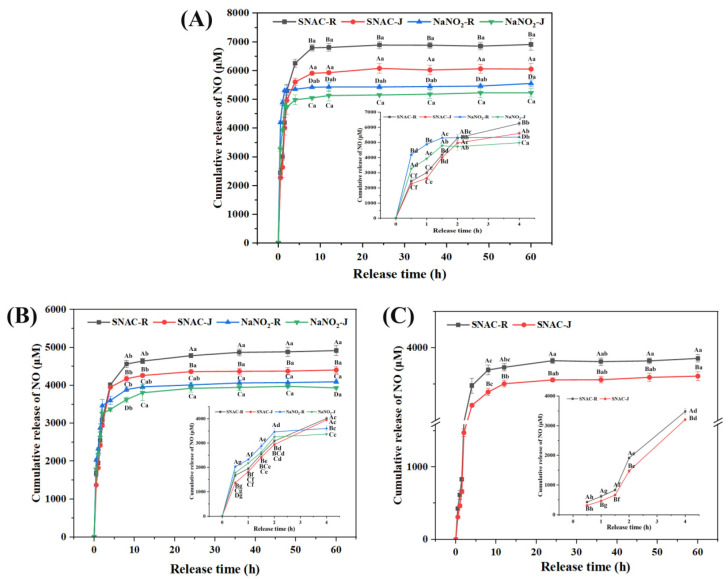
The cumulative release of NO in an *E. coli* solution and the content of NO inside *E. coli* cells after SNAC and NaNO_2_ treatment under different pH values. (**A**) pH 3; (**B**) pH 5; and (**C**) pH 7. R represents the cumulative release of the NO in an *E. coli* solution, and J represents the NO content in *E. coli* cells. Uppercase letters indicate significant differences (*p* < 0.05) between different treatments at the same pH. Lowercase letters indicate significant differences among different release times with the same treatment (*p* < 0.05).

The release behavior of NO from SNAC and NaNO_2_ during the antibacterial process was studied. The cumulative release of NO in *E. coli* suspension and the content of NO inside *E. coli* cells are shown in Figure 2. The cumulative release of NO ranged from 2443.3 μM to 6909.5 μM from SNAC and NaNO_2_ with a pH of 3 within 60 h. The rate of NO release from NaNO_2_ was faster than that from SNAC in the first 1.5 h (*p* < 0.05), followed by a slow release that reached equilibrium at about 2 h; meanwhile, SNAC maintained the release of NO into the medium until it achieved an equilibrium state around 8 h later. The content of NO inside *E. coli* cells with SNAC treatment was about 6052.1 μM, which was significantly higher than that of NaNO_2_ (5052.6 μM) at a pH of 3 (*p* < 0.05). This result was related to the higher ability of the NO release from SNAC, and the higher amount of NO might be related to the better antibacterial effect of SNAC. The release of NO ranged from 1371.1 μM to 4917.9 μM at a pH of 5. The rate of NO release from NaNO_2_ was faster than that from SNAC in the first 3 h (*p* < 0.05), followed by a slow release that reached equilibrium at about 3.5 h, whereas SNAC continued to release NO until it reached equilibrium at about 10 h. The content of NO inside *E. coli* cells with SNAC treatment was about 4404.9 μM, which was higher than that of NaNO_2_ (3936.1 μM) at a pH of 5 (*p* < 0.05), and the higher content of NO might endow the better antibacterial ability of SNAC. The release of NO ranged from 311.6 μM to 3855.4 μM in a pH of 7, SNAC was capable of persistently breaking down NO within the release medium, with the process being stable and reaching equilibrium approximately after 12 h. The cumulative release of NO from NaNO_2_ was not monitored at a pH of 7, mainly because NaNO_2_ hardly releases NO at a neutral pH. In one study, Carlsson et al. [31] previously investigated the production of NO from nitrite and the inhibition of bacterial growth in human urine at different pH levels. The results showed that human urine containing mildly acidified nitrite forms large amounts of NO and a strong inhibitory activity against the three most common pathogens of *E. coli*, *Pseudomonas aeruginosa*, and *Staphylococcus saprophyticus*, where the release of NO is enhanced by a decrease in pH, which is similar to the results of this study.

The content of NO inside *E. coli* cells after SNAC and NaNO_2_ treatment was less than that in the bacteria suspension (*p* < 0.05), and the reason for this was that the NO released by SNAC and NaNO_2_ was mainly in the *E. coli* suspension, where some of the NO entered the *E. coli* interior to exert an antibacterial effect. Secondly, it was not necessary for all of the NO to enter the bacteria cells.

### 3.4. Morphological Changes in E. coli Cells

**Figure 3:**
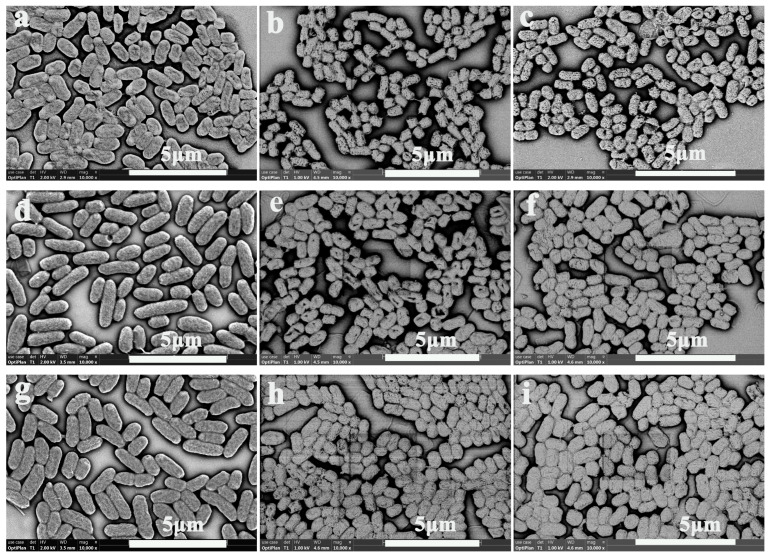
SEM of the *E. coli* cells after SNAC and NaNO_2_ treatment. (**a**–**c**) control, SNAC, and NaNO_2_, respectively, at a pH of 3; (**d**–**f**) control, SNAC, and NaNO_2_, respectively, at a pH of 5; and (**g**–**i**) control, SNAC, and NaNO_2_, respectively, at a pH of 7. Scale bar: 5 μm.

SEM was used to observe the morphological changes in the *E. coli* cells before and after SNAC and NaNO_2_ treatment. As can be seen from Figure 3, the cells in the control groups with a pH of 5 and 7 showed a full and short rod-like shape with relatively complete morphology. But the morphology of *E. coli* cells showed slight damage in the control group at a pH of 3 (Figure 3a), and this was mainly because the lower pH itself would have some inhibitory effect on *E. coli*. After SNAC and NaNO_2_ treatment (Figure 3b,c,e,f,h,i), some folds and collapses and plenty of holes appeared on the surface of the *E. coli* cells. Similar results were reported by Tian et al. [12], where they studied the bactericidal effects of bovine serum albumin microspheres (BSAMs) loaded with NaNO_2_ on *E. coli* and *S. aureus*. The SEM results indicate that, after treatment with BSAMs, significant folds and voids appear on the surface of bacterial cells, indicating that BSAMs loaded with NaNO_2_ have significant antibacterial effects.

The damage effect of both SNAC and NaNO_2_ was pH-responsive. The lower the pH, the more serious the damage to *E. coli* cells after SNAC and NaNO_2_ treatment. At a pH of 3, more cavities and damages appeared on the surface of cells after both SNAC and NaNO_2_ treatment. This reason could be explained by the fact that, at a lower pH, SNAC and NaNO_2_ derive more NO, reactive nitrogen, and reactive oxygen radical ions and their reactive radical ions, causing serious damage to bacterial cell walls and membranes [28], and that SNAC has a stronger damage effect than NaNO_2_ under a pH of 3, 5, and 7. This was mainly due to the amount of NO that was released from SNAC being higher than that of NaNO_2_, and the higher level of NO endowed SNAC with stronger antibacterial properties.

### 3.5. ROS Changes in E. coli Cells

**Figure 4:**
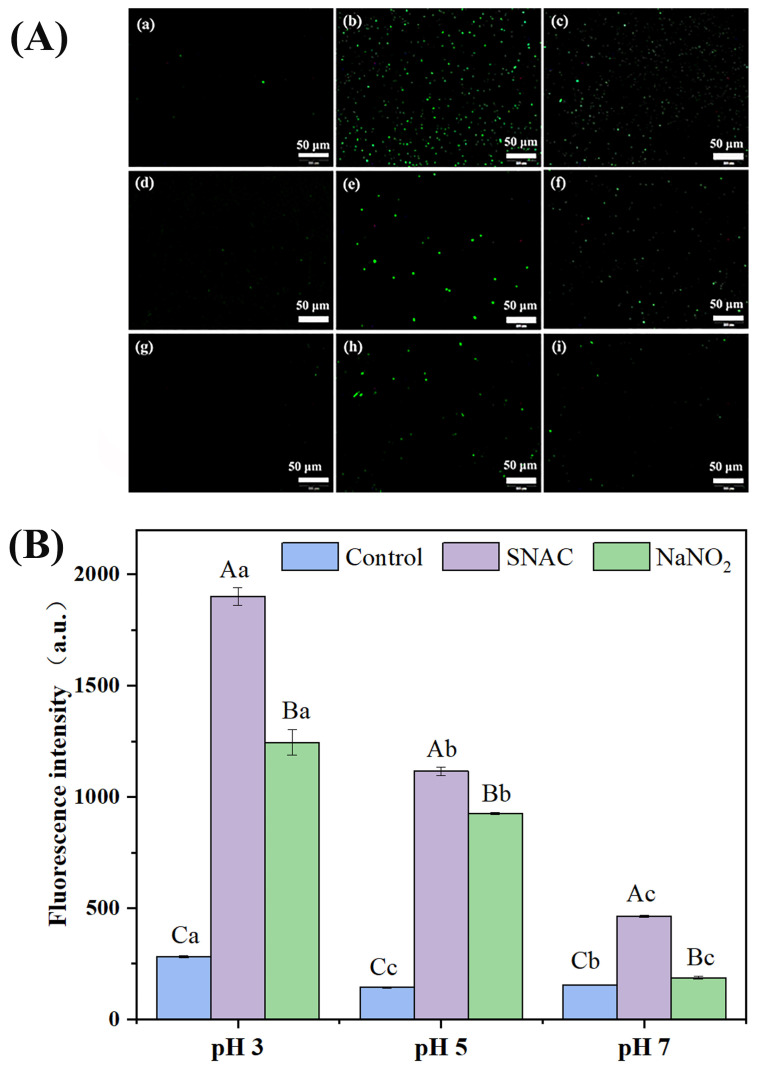
Fluorescence images of the ROS (**A**) and the level of ROS (**B**) in the *E. coli* cells after SNAC and NaNO_2_ treatments. (**a**–**c**) control, SNAC, and NaNO_2_, respectively, at a pH of 3; (**d**–**f**) control, SNAC, and NaNO_2_, respectively, at a pH of 5; and (**g**–**i**): control, SNAC, and NaNO_2_, respectively, at a pH of 7. Scale bar: 50 μm. Values are the means ± standard deviation of the triplicates. Uppercase letters indicate significant differences (*p* < 0.05) between different treatments at the same pH. Lowercase letters indicate significant differences among different pH values with the same treatment (*p* < 0.05).

ROS is a general term for oxygen radicals, which include the superoxide anion (O_2_^−^), hydrogen peroxide (H_2_O_2_), hydroxyl radical (·OH), and singlet oxygen (^1^O_2_) [32]. SNAC and NaNO_2_ can induce the production of ROS, which can destroy the permeability of the cell membrane and thus produce the antibacterial effect. From the fluorescence microscope picture (Figure 4A), it was clearly observed that there was no green fluorescence in the control groups with pH values of 5 and 7, but a weak fluorescence signal was produced in a pH of 3, which might be caused by the oxidative stress of the *E. coli* itself. In addition, a large number of cells showed green fluorescence after both SNAC and NaNO_2_ treatment at a pH of 3 (Figure 4A(b,c)), indicating more production of ROS. After both SNAC and NaNO_2_ treatments at a pH of 5 (Figure 4A(e,f)), some cells showed green fluorescence. After both SNAC and NaNO_2_ treatment at a pH of 7 (Figure 4A(h,i)), only a few cells showed green fluorescence. On the whole, the ROS production levels of SNAC and NaNO_2_ in *E. coli* were in the order of pH 3 > pH 5 > pH 7, indicating that the production level of ROS was pH-responsive. In addition, the production level of ROS in SNAC-treated *E. coli* cells was higher than that in NaNO_2_-treated ones.

In order to further detect the ROS levels in the *E. coli* cells, quantitative analysis was performed by a fluorescence enzyme spectrometer (Figure 4B). Through analyzing the fluorescence intensity values, it was found that, compared with the control groups of pH 3, 5, and 7 (282, 143, and 154 a.u.), the values of the SNAC-treated groups were 1901, 1116 and 464 a.u., respectively, and the values in the NaNO_2_-treated groups were 1246, 926, and 187 a.u., respectively. The fluorescence intensity increased with a decrease in pH, which indicates that the ROS production level is correlated with the pH. The fluorescence intensity of the SNAC-treated *E. coli* cells was higher than that of NaNO_2_, which indicates that the ROS induced by SNAC is significantly higher than NaNO_2_. On the whole, both SNAC and NaNO_2_ could induce the production of ROS, and the content of ROS far exceeded the amount that the *E. coli* cells could eliminate. Thus, excessive ROS caused damage to *E. coli*, which exerted antibacterial effects. When Li et al. [33] investigated the effects of NaNO_2_ and tea polyphenols on the cytotoxicity of carbon nanoparticles in fried pork, they found that carbon nanoparticles inhibit cell growth by increasing the level of cellular ROS, and the addition of NaNO_2_ induces the production of more ROS, which is consistent with the findings of this study.

### 3.6. RNS Changes in E. coli Cells

**Figure 5:**
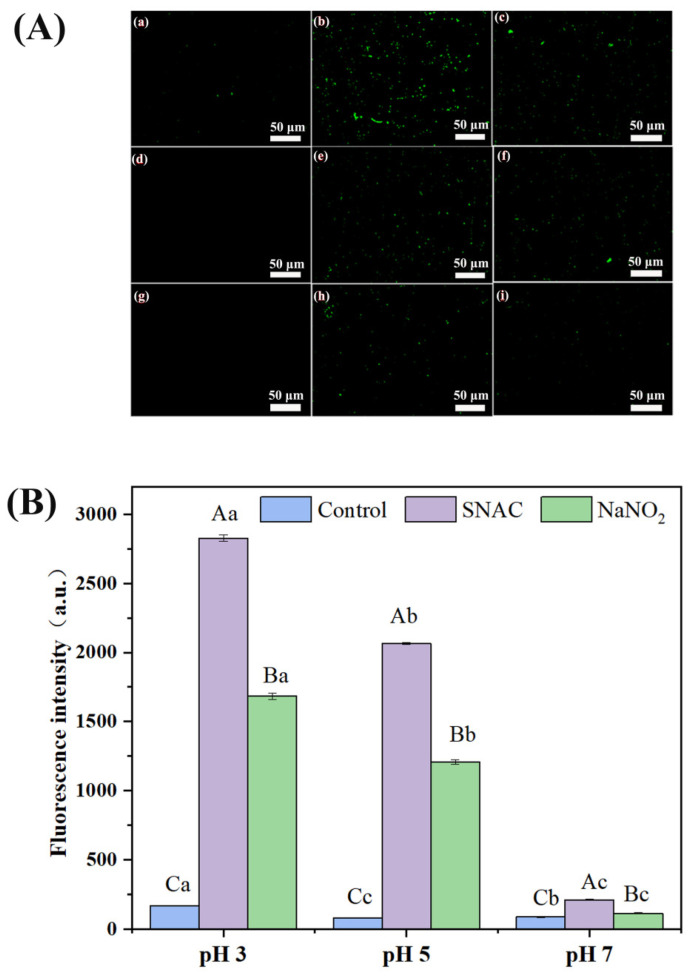
Fluorescence images of RNS (**A**) and the level of RNS (**B**) in *E. coli* after SNAC and NaNO_2_ treatment. (**a**–**c**) control, SNAC, and NaNO_2_, respectively, at a pH of 3; (**d**–**f**) control, SNAC, and NaNO_2_, respectively, at a pH of 5; and (**g**–**i**) control, SNAC, and NaNO_2_, respectively, at a pH of 7. Scale bar: 50 μm. Values are the means ± standard deviation of the triplicates. Uppercase letters indicate significant differences (*p* < 0.05) between different treatments at the same pH. Lowercase letters indicate significant differences among different pH values with the same treatment (*p* < 0.05).

RNS is a general term for NO and its by-products, which include nitrate (NO_3_^−^), nitrite (NO_2_^−^), peroxynitrite (ONOO-), nitrogen dioxide (NO_2_·), etc. [34]. An O_52_ fluorescent probe was used to measure RNS in the *E. coli* cells in this study. From the fluorescence microscope picture (Figure 5A), it was clearly observed that there was no green fluorescence in the control groups with a pH of 5 and 7, but a weak fluorescence signal was produced in the control group with a pH of 3 (Figure 5A(a)), which might be caused by the oxidative stress of *E. coli* itself. In addition, in the experimental groups, a large number of cells showed green fluorescence at a pH of 3 (Figure 5A(b,c)), indicating more production of RNS. Some cells showed green fluorescence at a pH of 5 (Figure 5A(e,f)), and only a few cells showed green fluorescence at a pH of 7 (Figure 5A(h,i)). The RNS production levels were in the order of pH 3 > pH 5 > pH 7 for both SNAC and NaNO_2_, indicating that the production level of RNS was pH-responsive. Likewise, the production level of RNS in the SNAC-treated cells was significantly higher than that in the NaNO_2_-treated groups.

The RNS level in the *E. coli* cells detected by fluorescence enzyme spectrometer is shown in Figure 5B. Compared with the control groups with pH values of 3, 5, and 7 (168, 79, 88 a.u.), the fluorescence intensity values in the SNAC-treated groups were 2827, 2064, and 213 a.u., respectively, and the values in the NaNO_2_-treated groups were 1684, 1207, and 114 a.u., respectively. The fluorescence intensity of the SNAC-treated groups was higher than that of the NaNO_2_-treated ones at the same pH, which indicates that the level of RNS induced by SNAC is significantly higher than NaNO_2_. In examining the impact of the NO/ROS/RNS cascade-releasing nanoplatforms on gas/photodynamic therapy/photothermal therapy/tumor immunotherapy, Ding et al. discovered that the cascade release of ROS and NO generates a greater quantity of RNS [35], and their findings align with the results in this study.

### 3.7. Lipid Peroxidation in E. coli Cells

**Figure 6:**
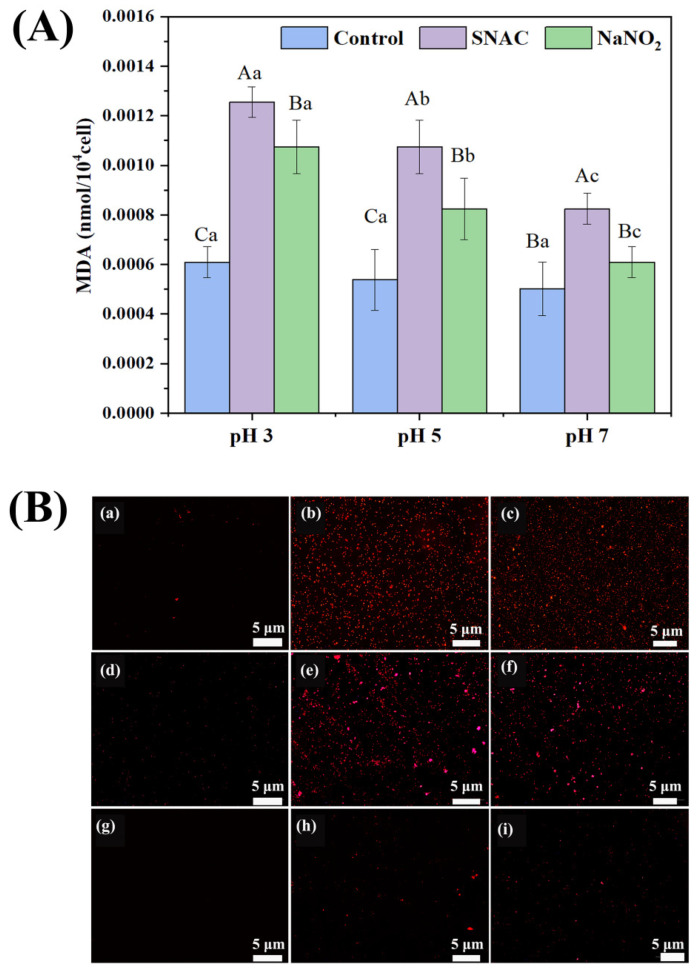
MDA content (**A**) and PI fluorescence staining (**B**) of *E. coli* after SNAC and NaNO_2_ treatment. (**a**–**c**) control, SNAC, and NaNO_2_, respectively, at a pH of 3; (**d**–**f**) control, SNAC, and NaNO_2_, respectively, at a pH of 5; (**g**–**i**) control, SNAC, and NaNO_2_, respectively, at a pH of 7. Scale bar: 5 μm. Values are the means ± standard deviation of the triplicates. Uppercase letters indicate significant differences (*p* < 0.05) between different treatments at the same pH. Lowercase letters indicate significant differences among different pH values with the same treatment (*p* < 0.05).

To substantiate the extent of damage inflicted by SNAC and NaNO_2_ on *E. coli* cells, an examination of the lipid oxidation in these cells was conducted. Lipid peroxidation serves as a marker of RONS damage, and its presence can be ascertained by measuring the levels of MDA. Figure 6A shows that the MDA contents of *E. coli* were 0.0007, 0.0013, and 0.0011 mmol/10^4^ cell; 0.0006, 0.0011, and 0.0008 mmol/10^4^ cell; and 0.0005, 0.0010, and 0.0006 mmol/10^4^ cell for the control, SNAC, and NaNO_2_ groups at pH values of 3, 5, and 7, respectively (*p* < 0.05). The MDA content in the SNAC and NaNO_2_ groups markedly surpassed that in the control group at the same pH, indicating that the damage to *E. coli* cells by SNAC and NaNO_2_ was considerably greater compared to the control groups. With a decrease in the pH, higher lipid peroxidation of cells occurred, which clarified the harm inflicted upon *E. coli* by RONS following treatments with SNAC and NaNO_2_.

### 3.8. Fluorescence Staining Analysis of E. coli Cells

PI can pass through the damaged cell membrane to stain the nucleus and produce red fluorescence [36], so PI is often used to assess the changes in the membrane permeability of bacterial cells [37]. As shown in Figure 6B, the control groups at a pH of 5 and 7 had almost no red fluorescence, whereas a little bit of red fluorescence appeared in the control group at a pH of 3, which was due to the fact that the lower pH itself would have some damaging effect on the *E. coli* cells. After SNAC and NaNO_2_ treatment (Figure 6B(b,c,e,f,h,i)), the cells showed a notable amount of red fluorescence. Among these, almost all the cells showed red fluorescence at a pH of 3 (Figure 6B(b,c)), indicating serious cell membrane damage. Under a pH of 5 (Figure 6B(e,f)), half of the cells showed red fluorescence. Under the treatment of a pH of 7 (Figure 6B(h,i)), only a small number of cells showed red fluorescence, signifying that most of the cells remained in a relatively intact state. The fluorescence intensity of PI in the SNAC-treated cells was higher than that in the NaNO_2_-treated ones, indicating that SNAC causes more extensive damage to the cell membranes of *E. coli*. On the whole, both SNAC and NaNO_2_ exerted antibacterial effects by changing the permeability of bacterial cell membranes, especially SNAC.

### 3.9. Application of SNAC and NaNO_2_ in Beef Sausages

#### 3.9.1. Total Colony Counting

**Figure 7:**
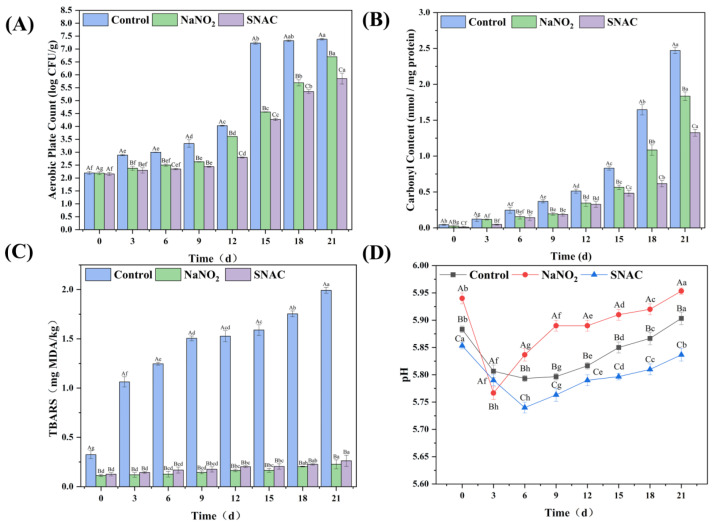
Effects of SNAC and NaNO_2_ on the total colony count (**A**), carbonyl content (**B**), TBARS (**C**), and pH (**D**) of the sausages during storage. Uppercase letters indicate significant differences (*p* < 0.05) among different treatments at the same storage time. Lowercase letters indicate significant differences (*p* < 0.05) among different storage times with the same treatment.

Figure 7A shows the changes in the total colony counts of sausages under a 4 °C storage. The total colony count increased with an increase in storage times (*p* < 0.05), and the total colony count in the experimental group was significantly lower than that in the control group (*p* < 0.05), which indicates that the bacterial inhibition effects of SNAC and NaNO_2_ are significant. Moreover, SNAC had a stronger inhibitory effect than NaNO_2_, which might be because SNAC released more NO, thus conferring a stronger inhibitory activity [38]. On Day 15, the total colony counts of the control group exceeded 7 log CFU/g. In general, 7 log CFU/g is used as the upper limit for meat products [39], and exceeding this limit indicates that the meat is spoiled and inedible. On Day 21, the total colony count of the SNAC group was 5.85 log CFU/g and the NaNO_2_ group was 6.70 log CFU/g (*p* < 0.05), which is still less than 7 log CFU/g, suggesting that SNAC and NaNO_2_ can effectively prolong the shelf life of sausages, especially SNAC. However, in the study of Shpaizer et al. [29], when they prepared beef sausages containing 150 ppm (2.17 mM) NaNO_2_ and SNAC and measured the total aerobic bacterial load at the end of the shelf life, they found that the bacterial load of SNAC-treated sausage was almost similar to that of nitrate. The different results might be the different amounts of SNAC and NaNO_2_ used.

#### 3.9.2. Carbonyl Content

Lipid oxidation and the attack of ROS and metal catalysts on functional groups located on amino acid side chains can lead to the formation of different protein radicals and hydroxyl derivatives, which then cause protein carboxylation. In general, the higher carbonyl content indicates the more severe protein oxidation. As shown in Figure 7B, the carbonyl content of sausage increased significantly (*p* < 0.05) with the extension of storage time, and the carbonyl content of the experimental group was significantly lower (*p* < 0.05) than that of the control group. The reason for this might be that SNAC and NaNO_2_ can release NO to bind myoglobin and form a stable nitroso myoglobin (NOMb), which inhibits the release of heme iron, reduces the generation of free radicals, and retards protein oxidation [40]. Meanwhile, as the NO release of SNAC was found to be higher than NaNO_2_, the carbonyl content of SNAC was lower than NaNO_2_, indicating that SNAC has better antioxidant properties. In the study of Feng et al. [41], they studied the effect of different concentrations of NaNO_2_ on the protein oxidation of cooked sausages, and the results showed that NaNO_2_ could inhibit the conversion of free amino groups to carbonyl groups such that the carbonyl content decreased, which was similar to our results.

#### 3.9.3. TBARS Value

The TBARS value is the prevalent benchmark for assessing the extent of lipid oxidation in meat products. The higher the TBARS value, the more serious the lipid oxidation. As shown in Figure 7C, the TBARS value of the sausages increased with the storage days, while the TBARS value of the experimental group increased slower than the control, which indicates that SNAC and NaNO_2_ effectively inhibit lipid oxidation (*p* < 0.05). Meanwhile, the TBARS values of the NaNO_2_ group were slightly lower than those of the SNAC group, but there was no significant difference (*p* > 0.05). Bruna Fernandes Andrade et al. [42] found that the TBARS value of NaNO_2_-treated recombinant ham was significantly lower than that of equimolar SNAC-added (50 mg NEq/kg) recombinant ham. According to Kanner and Juven [43], under anaerobic conditions of 2 °C or 27 °C, the TBARS value of a comminuted turkey meat product was very low, and no changes in the SNAC- or NaNO_2_-treated samples were found after 14 days of storage.

#### 3.9.4. pH

As can be seen from Figure 7D, the trend of the pH of the sausages in the SNAC, NaNO_2_, and control groups was the same, all of them first decreased and then increased (*p* < 0.05). This might be because, during the pre-storage period, the pH of the sausages decreased due to the high moisture content and the high microbial activity, which decomposed carbohydrates and fats and produced organic acids, such as free fatty acids, lactic acid, and other acidic substances [44]. With the prolongation of storage time, the endogenous or exogenous enzymes derived from microorganisms degraded proteins to produce alkaline substances such as amines, and the pH of the sausages increased [45]. In addition, on Day 0, the order of the initial pH values was as follows: SNAC < Control < NaNO_2_ (*p* < 0.05). This might be because the aqueous solution of NaNO_2_ was alkaline and the aqueous solution of SNAC was acidic. Meanwhile, under acidic conditions, SNAC was able to exert its bacteriostatic effect better, which is consistent with the total colony count results.

#### 3.9.5. Color

Table 1 reflects the color changes in the sausages. In general, the a* value is an important index to assess the color of sausage, and it can be seen that the a* value of the sausages added with SNAC and NaNO_2_ was significantly higher than that of the control group (*p* < 0.05), indicating that SNAC and NaNO_2_ could improve the red color of sausages. But no substantial variation was observed between these two experimental groups (*p* > 0.05). However, the a* value decreased with an increase in storage duration. This phenomenon could be explained by the fact that, at the early stage of the experiment, NO bonded with myoglobin and formed heat-stable nitroso myoglobin (NOMb), and at the later stage, NOMb was gradually decomposed by light, and the red color was weakened [16].

## 4. Conclusions

In conclusion, the antibacterial activity of SNAC and NaNO_2_ on *E. coli* was pH-responsive (pH 3, 5, and 7); specifically, the lower the pH, the higher the antibacterial effect. Under the same pH, SNAC showed better antibacterial activity on *E. coli* than NaNO_2_. The morphology of *E. coli* was changed after SNAC and NaNO_2_ treatments and numerous holes appeared on the cell surface, along with the permeability of cellular membrane increasing, thus inhibiting the growth of *E. coli*. Likewise, SNAC and NaNO_2_ induced the increase in RONS in *E. coli*, and the value in SNAC-treated *E. coli* was significantly higher than that of NaNO_2_. Moreover, the increase in lipid oxidation confirmed the damage caused by RONS to *E. coli* after SNAC and NaNO_2_ treatments. When SNAC and NaNO_2_ were applied in beef sausages, SNAC showed better antibacterial and anti-protein oxidation effects than NaNO_2_. Overall, these results can provide some references for SNAC to replace NaNO_2_ in meat products.

## Figures and Tables

**Table 1:** Effects of SNAC and NaNO_2_ on the a* of sausages during storage.

Time (Days)		Treatment	
	Control	SNAC	NaNO_2_
0	4.76 ± 0.56 ^Ba^	13.14 ± 0.77 ^Aa^	12.74 ± 1.20 ^Aa^
3	4.72 ± 0.94 ^Ca^	13.09 ± 0.62 ^Aa^	11.72 ± 0.76 ^Bb^
6	4.62 ± 0.82 ^Ba^	11.95 ± 1.20 ^Ab^	11.64 ± 0.63 ^Ab^
9	4.57 ± 0.63 ^Ba^	11.79 ± 1.17 ^Ab^	11.38 ± 0.78 ^Ab^
12	4.42 ± 0.90 ^Ba^	11.72 ± 1.11 ^Abc^	11.28 ± 0.59 ^Abc^
15	4.04 ± 0.78 ^Bab^	11.63 ± 0.45 ^Abc^	10.82 ± 0.73 ^Abcd^
18	3.26 ± 0.78 ^Cb^	11.14 ± 0.48 ^Abc^	10.35 ± 0.38 ^Bcd^
21	2.26 ± 0.52 ^Bc^	10.64 ± 0.88 ^Ac^	10.00 ± 0.79 ^Ad^

Uppercase letters indicate significant differences (*p* < 0.05) among different treatments at the same storage time. Lowercase letters indicate significant differences (*p* < 0.05) among different storage times with the same treatment.

## Data Availability

The original contributions presented in the study are included in the article, further inquiries can be directed to the corresponding author.
